# Pediatric haematopoiesis and related malignancies

**DOI:** 10.3892/ol.2021.12853

**Published:** 2021-06-07

**Authors:** Mingwei Jin, Shumei Xu, Qi An

Oncol Lett 14: 10-14, 2017; DOI: 10.3892/ol.2017.6106

Subsequently to the publication of the above review, an organization named The Pulsenotes Team contacted the Editorial Office to explain that the figure printed in the article (“Figure 1: Haematopoeisis in humans”) had been extracted from their stock library, and had been used without the authors having solicited permission from them to use it. After our contacting The Pulsenotes Team, they were satisfied that this matter would be resolved by the figure being credited to them as its proper source.

Therefore, the figure is reprinted opposite, now featuring an acknowledgement to The Pulsenotes Team as the source of this figure (shown in bold). Furthermore, text for an Acknowledgments section for this paper has been added as follows:

**Acknowledgments**

Note that the figure used in this review was reproduced with kind permission of The Pulsenotes Team (www.pulsenotes.com).

These corrections have also been added to the published version of the paper. Note that the authors were also consulted, but the Editorial Office did not receive a timely response from them. The Editor of *Oncology Letters* regrets that this review was published without a proper acknowledgement being made to The Pulsenotes Team as the source of the figure, and the Journal is grateful to them for raising this matter with us as a concern. Furthermore, we apologize to The Pulsenotes Team for the inconvenience caused.

## Figures and Tables

**Figure 1.**
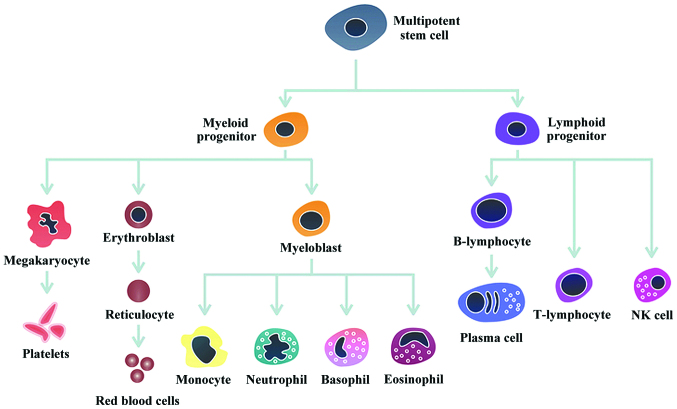
Haematopoiesis in humans. **This Figure is reproduced with kind permission of The Pulsenotes Team (www.pulsenotes.com).**

